# Influence of Material Microstructures in Micromilling of Ti6Al4V Alloy

**DOI:** 10.3390/ma6094268

**Published:** 2013-09-24

**Authors:** Aldo Attanasio, Marcello Gelfi, Annalisa Pola, Elisabetta Ceretti, Claudio Giardini

**Affiliations:** 1 Department of Mechanical and Industrial Engineering, University of Brescia, Via Branze 38, Brescia 25123, Italy; E-Mails: marcello.gelfi@ing.unibs.it (M.G.); annalisa.pola@ing.unibs.it (A.P.); elisabetta.ceretti@ing.unibs.it (E.C.); 2 Department of Engineering, University of Bergamo, Via Marconi 5, Dalmine (BG) 24044, Italy; E-Mail: claudio.giardini@unibg.it

**Keywords:** titanium alloy, microstructure, micromilling, cutting forces

## Abstract

In the most recent decades the introduction of unconventional machining processes allowed the development of micromachining techniques. In this work, the influence of material microstructures on the micromilling process was investigated. Ti6Al4V alloy was selected as workpiece material since it is a very common material for micro applications and because its duplex microstructure can be easily changed by proper thermal treatments. Four different microstructures (namely bimodal, fully equiaxed, fully lamellar and mill annealed) were obtained through recrystallization annealing treatments carried out at different times and temperatures. The mechanical properties of the samples were assessed by microhardness measurements. Nano-indentations were also performed on single grains to understand how the different hardness of phases and structures present in the Ti6Al4V alloy can affect the micromilling process. Microchannels using two flute flat end mills with a diameter equal to 200 µm were realized on the treated samples. Two different feed-per-tooth values were used during the tests. Cutting force, channel shape and burr dimension were investigated. Morphological and energy dispersive spectroscopy (EDS) analyses were performed on tools by means of a scanning electron microscope (SEM): in this way the phenomena mainly influencing the tool status were also identified. Lower cutting forces and reduced tool wear were observed when working fully lamellar microstructures compared to the other ones.

## 1. Introduction

Nowadays, the term “micro” is very common in many manufacturing fields. In fact, in the most recent decades the introduction of new manufacturing technologies finalized to ultra-precision production, allowed the beginning and the development of micromachining processes. Despite the novelty, state of the art and trends of these working techniques had already been considered and published by Taniguchi [1] in 1983.

As reported by Masuzawa in [2], micromachining processes can be classified on the basis of the phenomena that cause the material removal. In fact, to manufacture a micro component it is possible to use mechanical force, e.g., microcutting, microgrinding and ultrasonic machining; material melting and vaporization, e.g., micro electron discharge machining (μEDM), electron beam machining (EBM) and laser beam machining (LBM); material ablation, e.g., LBM; chemical or electro-chemical dissolution of material in liquids, e.g., electro chemical machining (ECM); plastic deformation, e.g., microforming, solidification of liquid or paste materials in a die, e.g., microcasting and microinjection molding; layer by layer solidification, e.g., stereolitography; material recomposition from metal ion deionized in an electrolyte liquid, e.g., electroforming.

An improved classification of micromachining processes is reported in [3], where Alting *et al.* discussed the problems dealing with micro component development, highlighting the interconnections amongst part design, material type, manufacturing process, part assembly and product control/measurement in the process chain.

From an industrial point of view, there are several and different fields where components with micro features with dimensions ranging from 1 μm up to 500 μm [2] (holes, pins, slits, grooves, pockets, 3D shaped surfaces, textures) are needed. Micro products are used in the mechanical industry [4,5] (e.g., watch gears, micro transmission, micro dies); in the electronic industry [4,6] (e.g., micro sensors, micro actuators); in the aerospace industry [7] (e.g., effusion cooling holes in nozzle and turbines blades, fuel injector nozzles); in the automotive field [4] (e.g., fuel injection nozzle) and in the biomedical field [4,8] (e.g., implants, surgery devices and lab on chips).

The selection of the most suitable manufacturing process (microcutting, micromilling, microEDM, LBM, ECM, *etc.*) is a relevant issue especially in micro scale, since the material removal phenomena strongly influence the part functionality. Geometrical accuracy, surface integrity, maximum obtainable shape, aspect ratio and material batch dimension are some examples of characteristics that must be taken into account when selecting the best performing micro manufacturing process.

The micromilling process has interesting advantages over other micromachining processes (LASER, EDM, EBM). It is a flexible and versatile process, characterized by short set-up times and able to realize complex shapes while working different materials. For these characteristics, in recent years, this manufacturing technique has been studied by several researchers so improving the understanding of the phenomena involved with the process itself.

When performing microcutting processes (microturning, micromilling and microdrilling), it is very important to identify the minimum chip thickness under which the chip does not form. The minimum chip thickness is related to the ratio between the depth of cut and the tool edge radius. This ratio influences the final part quality and the material removal mechanism can be one of cutting, ploughing, and slipping as demonstrated by Dornfeld *et al.* [9].

Researches related with other aspects of microcutting processes can be found in the literature demonstrating the interest of scientific and industrial communities on these manufacturing processes. Bissacco *et al.* [10] analyzed the surface topography generated on hardened steel by using ball and flat end micromills focusing their attention on the size effects on surface generation. Altintas *et al.* [11] investigated the mechanics of micromilling by using round edge tools. Also a cutting force prediction model, based on slip line field theory, was proposed and experimentally tested. Zaman *et al.* [12] introduced a 3D analytical model for force prediction in micromilling. The theoretical chip area, at any specific angular position of the tool cutting edge, was defined taking into account the cutting edge path and the cutting forces. The FEM approach coupled with experimental observations was utilized by Özel *et al.* [13], Afazov *et al.* [14] and Jin [15] to study forces in micromilling process.

In [16] Uhlmann *et al.* reported a study focused on the end micromill geometry optimization through the analysis of cutting forces and tool strain, demonstrating that the geometries of today’s mills are not appropriate for the type of load they have to support. Aramcharoen in [17] and Giorleo in [18] investigated the influence of tool coatings in micromilling and microdrilling processes respectively, showing that both tool wear and part quality can be improved by selecting the most appropriate coating.

Other researches have been focused on final part quality, on material type and on the microcutting process studying the influence of process parameters such as lubrication type [19], feed rate, cutting speed and depth of cut [20,21].

The aim of the present study is to contribute to the field of the micromachining technique by analysing the influence of material microstructure on the micromilling process. In fact, when considering microcutting processes, the uncut chip thickness and/or the feed-per-tooth often tend to be of the same order of magnitude as the grain size.

Ti6Al4V alloy was selected as testing material, since it is widely used for manufacturing micro parts and because its microstructure can be easily changed by proper thermal treatments. Ti6Al4V is a two phase alloy since it retains alpha (hexagonal close-packed) and beta (body-centred cubic) crystalline forms at room temperature [22,23]. The transformation temperature from α to β is called transus temperature, which for this alloy is about 995 °C [24]. Depending on recrystallization annealing time and temperature, different microstructures and mechanical properties can be obtained, namely: bimodal (duplex) microstructure, containing equiaxed primary α in a lamellar α + β matrix, which has an excellent combination of mechanical properties; fully lamellar microstructure, having high toughness but low ductility; fully equiaxed microstructure, with fairly good strength and ductility.

A common but less defined microstructural condition is the so-called mill-annealed structure, which is the result of cooling after plastic deformation, without any recrystallization annealing.

As received Ti6Al4V bar in mill annealed condition was thermally treated obtaining the four above mentioned microstructures. Micro channels were realized on the samples by using a two flute flat end mill with a diameter of 200 μm and setting two different feed-per-tooth.

Cutting forces were acquired by using a load cell, while burr dimensions, channel sections and tool aspect were analysed by using SEM and an optical microscope. Statistical methods (ANOVA analysis) were applied to these experimental data in order to assess the influence of material microstructures and cutting parameters on the micromilling of Ti6Al4V alloy.

## 2. Experimental Procedure

### 2.1. Material Microstructures Preparation

**Figure 1:**
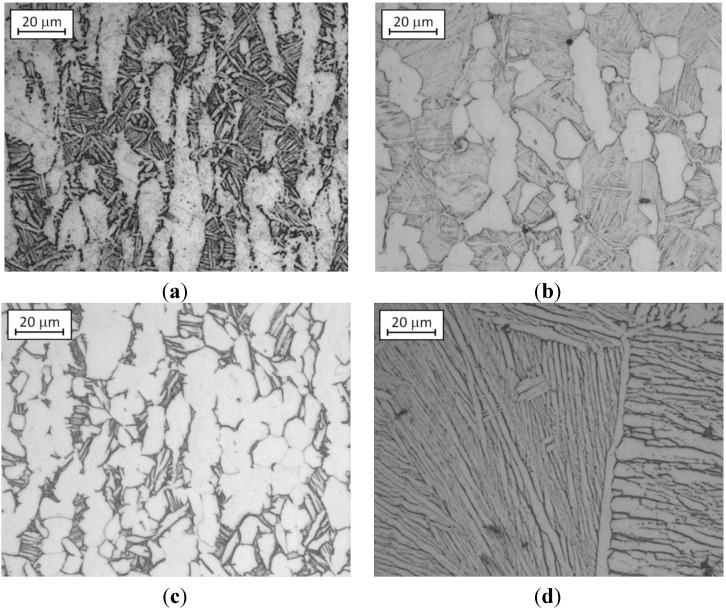
Optical microscope images of Ti6Al4V microstructures: (**a**) mill annealed; (**b**) bimodal; (**c**) fully equiaxed and (**d**) fully lamellar.

The as received microstructure of Ti6Al4V bar (20 mm diameter) was revealed on a mirror polished sample by Kroll’s Reagent etching (6 mL HNO_3_, 2 mL HF in 100 mL distilled water) applied for 20 s, which darkens the β phase and leaves unetched the α phase. The sample observed with the optical microscope shows the presence of a mill annealed microstructure (Figure 1a). This microstructure was modified according to [23] by specific thermal treatments.

Considering the high reactivity of Ti alloys and the risk of forming excessive oxidation of the sample surface, the thermal treatments were carried out in a silica tube furnace under argon shielding. The tube was purged for 20 min without the sample at a flow rate of 2 L/min, which was reduced to 0.5 L/min during sample heating and cooling down.

Three different thermal treatments were carried out and three new microstructures were obtained: bimodal microstructure (Figure 1b): sample was heated at 955 °C (about 45 °C under the β-transus), soaked for 60 min and furnace cooled; fully equiaxed microstructure (Figure 1c): sample was heated at 925 °C, soaked for 240 min and furnace cooled. Figure 1b shows that at the grain boundary of equiaxed α grains there is still some untransformed β lamellae; fully lamellar microstructure (Figure 1d): sample was heated at 1035 °C (about 35 °C above the β-transus), soaked for 30 min and cooled down in the switched-off furnace (averaged cooling rate of 4–5 °C/min).

### 2.2. Microstructures Characterization

Optical images, collected at magnification of 400×, were analyzed by Leica QWin Image Software (Leica Microsystems GmbH, Wetzlar, Germany) to assess the different volume fractions of equiaxed α and lamellar grains present in the bimodal, fully equiaxed and mill annealed microstructures. The results reported in Table 2 are an average of at least 10 images for each sample.

The mechanical properties of Ti6Al4V microstructures were measured by a Shimadzu Vickers microhardness tester (Shimadzu Corporation, Kyoto, Japan), using a load of 4.9 N and performing 60 measurements for each microstructure.

**Figure 2:**
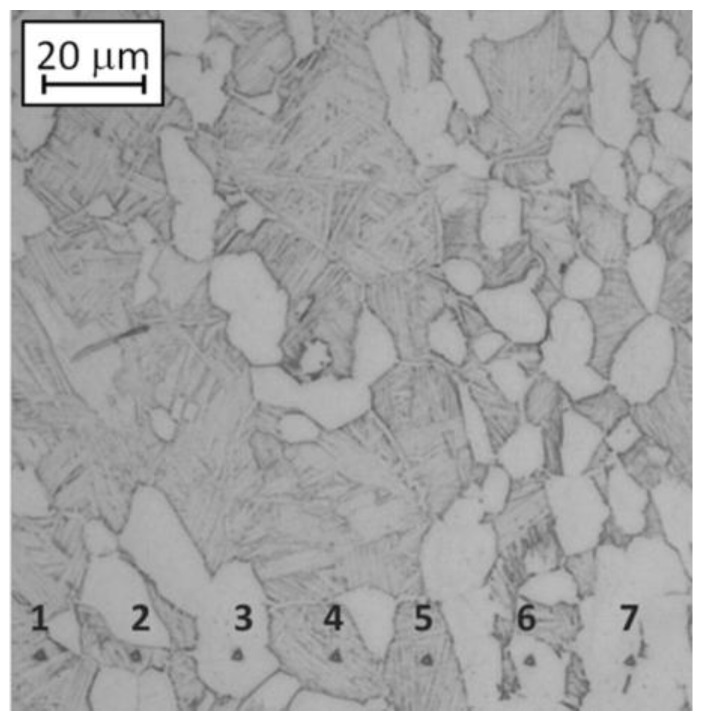
Example of nano-indentations performed with a load of 20 mN on a bimodal etched sample.

Nano-indentation tests were carried out by using a Table Top Nano-Indentation Tester (TTX-NHT) by CSM Instruments (CSM Instruments SA, Peseux, Switzerland). The maximum load was set at 20 mN to maintain the indentation size of the order of 2 microns. A row of 60 indentations was automatically performed on the unetched sample. Afterwards, the microstructure was revealed and only the indentations which were completely inside the equiaxed α grains or inside the lamellar ones were considered for the results (Figure 2): in this way, the hardness of each type of grain was assessed.

### 2.3. Micromilling Tests

**Figure 3:**
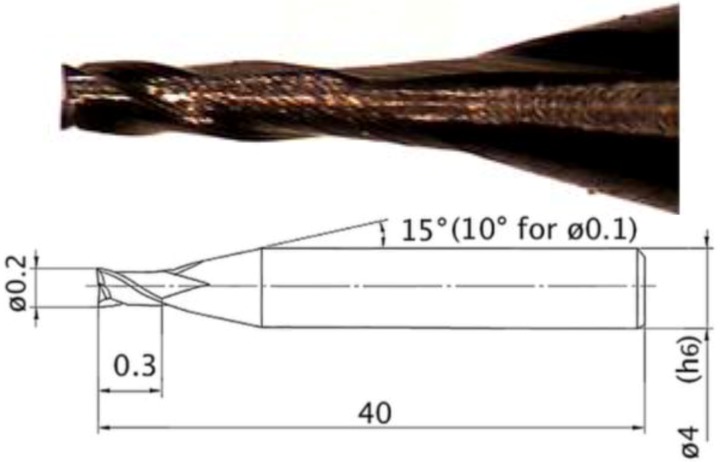
Mitsubishi MS2SSD0020 mill.

The experimental tests consisted of machining micro channels on the treated samples by using flat end mills with two flutes (Figure 3). Design of experiment (DOE) technique was applied for planning the experiments. The micro channels were realized with a Kern Pyramid Nano as described in [25]. The two flute flat end mills had a diameter of 200 μm, a length of cut equal to 300 μm and were made of tungsten carbide (WC-Co) with a titanium aluminum nitride [(Ti, Al)N] coating suitable for milling hardened materials.

The cutting velocity was set equal to 25,000 rpm (15.7 m/min) while two different feed-per-tooth were tested (0.5 and 1.5 μm/tooth). The depth of cut was constant and set equal to 50 μm. Dry cutting was taken into account to analyze the worst working conditions to exalt the phenomena involved on the cutting operation, to increase the cutting force values and to accelerate tool wear. Moreover, the aim of the research was to highlight the influence of the material microstructure on the process and, although beneficial in terms of built up edge and burr reduction, the use of lubricant during the experimental tests can mask the material microstructure effect on the process.

**Figure 4:**
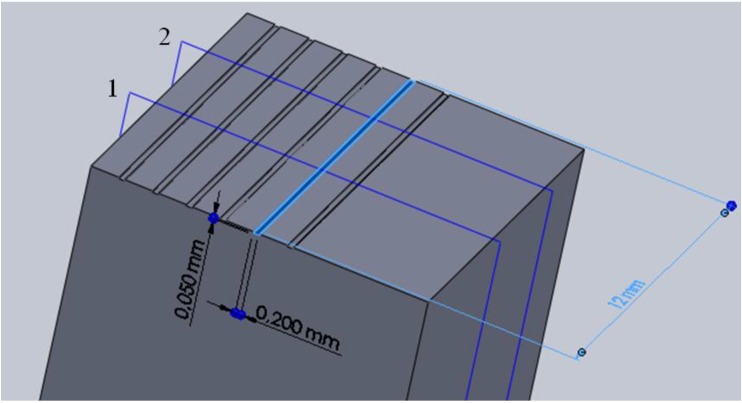
Sample and micro channel geometry.

The micro channel geometry is a straight slot with a rectangular section realized in a single pass and characterized by a width equal to 200 μm (the mill diameter), a depth of 50 μm, and a length of 12 mm (the sample length), as shown in Figure 4.

Before realizing the micro channels, the top side of each sample was lightly milled to guarantee flat and uniform surface. For each test a new micro end mill was utilized and three repetitions for each working condition were carried out.

The influence of material microstructure and feed rate were evaluated by comparing the detected cutting forces, the micro mill status after the cut, the micro channels shape and the burrs.

To detect micromilling forces a six-axis force/torque sensor (ATI Mini40 SI-80-4) (ATI Industrial Automation, Apex, NC, USA) made of silicon strain gauges was utilized. It is characterized by a sensing range of Fx, Fy: 80 N and Fz: 240 N and a resolution of Fx, Fy: 1/200 N and Fz: 1/100 N. For the analysis of micro mill geometry and micro channel shapes and dimensions, an optical microscope and a scanning electron microscope LEO EVO 40 (Carl Zeiss, Oberkochen, Germany) equipped with an Oxford energy dispersive spectroscopy (EDS) probe (Oxford Instruments, Abingdon, UK) for elemental analysis were utilized. The effect of the different parameters and their interaction was evaluated by ANOVA (analysis of variance between groups) analysis.

## 3. Results and Discussions

### 3.1. Microstructures Characterization

Table 1 reports the mean values of the measured microhardness on the Ti alloy microstructures and the corresponding standard deviations. The fully lamellar sample shows the lowest hardness and the highest scatter of the data because, as well known, coarse lamellae and/or grain size decrease the material strength.

**Table 1:** Mean and standard deviation of microhardness results for the different examined microstructures.

microHV	Mill annealed	Bimodal	Fully equiaxed	Fully lamellar
Avg.	386	411	397	356
Std.dev.	18	15	24	45

Table 2 shows the percentage of equiaxed and lamellar grains present in the bimodal, fully equiaxed and mill annealed microstructures and the corresponding hardness measured by nano-indentation. These hardness values are significantly higher with respect to those of micro Vickers. This can be explained by considering the indentation size effect (ISE), *i.e.*, the variation of the hardness with applied load [26], or the fact that the samples surface was strain hardened during mechanical polishing and that nano-indentations, having a penetration depth of a few hundreds of nanometers, can be affected by this hardening.

Nevertheless, some interesting observations can be pointed out. The first is that the lamellar grains are much softer than the equiaxed ones. This is mainly a consequence of element partitioning effects [27]. For the same reason the hardness of equiaxed α grains is maximum in the bimodal microstructure where its volume fraction is minimum. These considerations show that the low Vickers microhardness measured on a fully lamellar sample is a consequence of both element partitioning effects and of the coarse size of α lamellae.

**Table 2:** Volume fractions and nano-hardness of equiaxed and lamellar grains for the different microstructures.

Grain type	Mill annealed		Bimodal		Fully equiaxed	
	%	Hardness	%	Hardness	%	Hardness
Equiaxed α grain	43	499 ± 61	37	543 ± 46	87	481 ± 61
Lamellar (α + β) grain	57	397 ± 51	63	413 ± 30	13	417 ± 48

### 3.2. Cutting Forces

**Figure 5:**
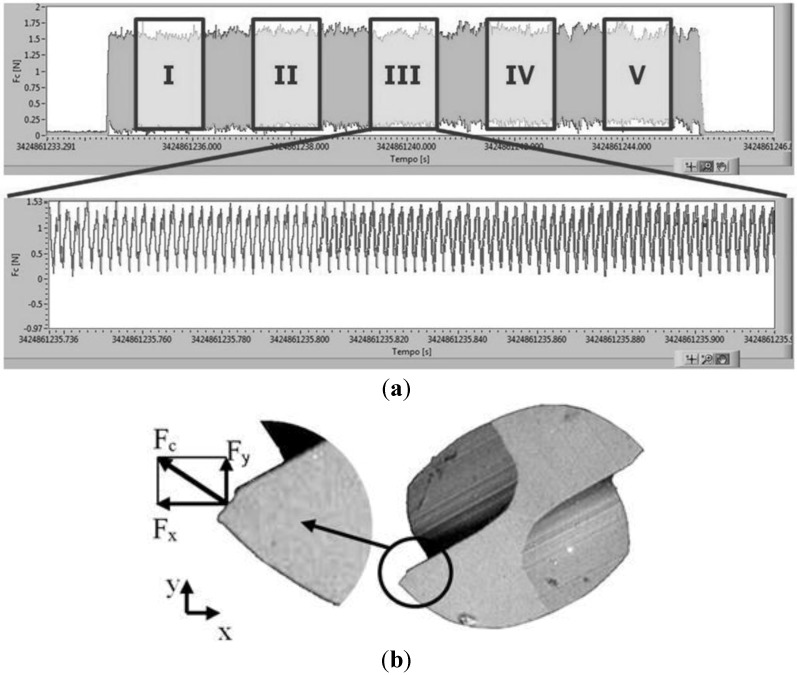
(**a**) Cutting force signal from Nationa Instrument LabVIEW based data acquisition system (DAQ) (bimodal microstructure and feed-per-tooth equal to 0.5 μm/tooth) and (**b**) process forces schematization.

Figure 5a shows an example of the cutting force signal coming from the data acquisition system (DAQ) when realizing a channel. The value of cutting force (F_c_) is obtained as a vector sum of the force components along the X (F_x_) and Y (F_y_) directions (Figure 5b). The force component along the Z axis was neglected since its value was always lower than the F_x_ and F_y_, ranging from 0.2 N up to 0.3 N.

Because of the high working frequency (833 Hz) and the variability of the signal, five windows equally distributed along the channel length were selected for the force analysis as shown in Figure 5a. For each window the maximum value of the cutting force was calculated as the mean value of the 10 highest picks. In this manner 15 force values for each cutting condition were obtained.

**Figure 6:**
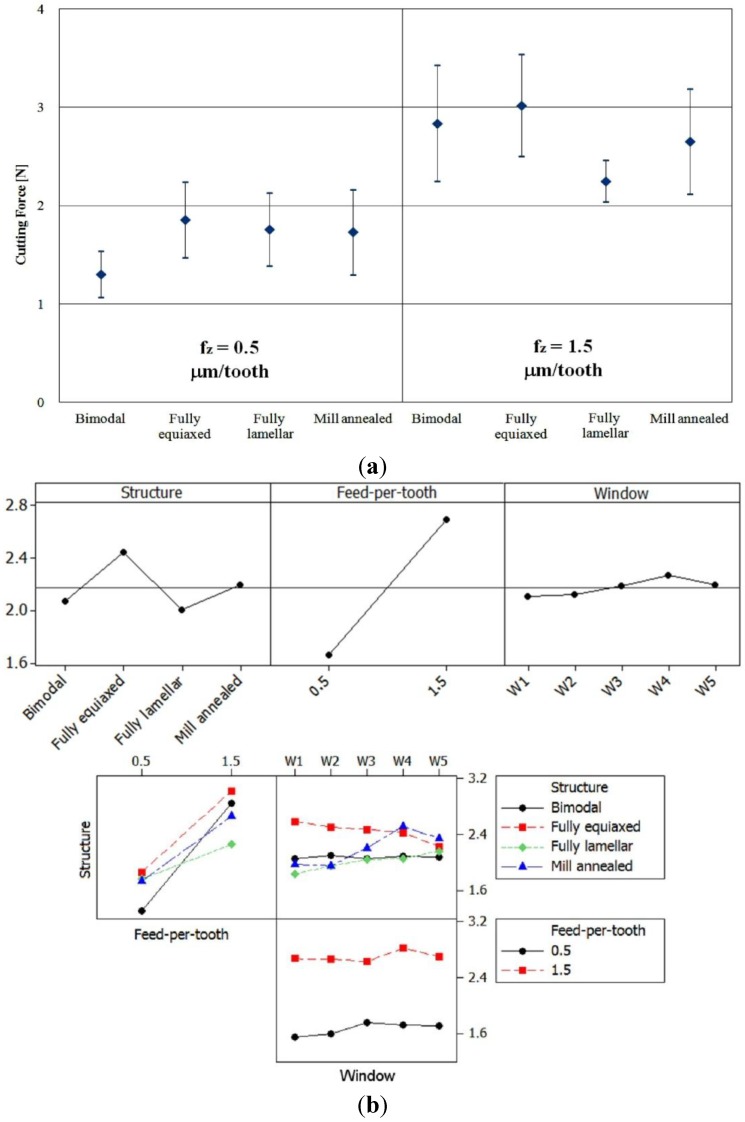
(**a**) Mean value and standard deviation of cutting forces measured for each cutting condition and (**b**) main effects and interaction plots for the cutting forces.

In particular, three factors were considered for the DOE analysis: material microstructure, feed-per-tooth, and windows position, while studying their influence on the cutting force. Figure 6a reports the measured forces for each cutting condition, while Figure 6b shows the main effects and the interaction plots of the considered factors: it is evident how material microstructure, feed-per-tooth, and their interaction affect the cutting force while no influence can be identified for the sampling windows. In particular, in the case of feed-per-tooth equal to 1.5 μm/tooth, the cutting force values are strongly related to the microstructure hardness. The lowest cutting force was measured when milling the soft lamellar microstructure, while the highest cutting forces were recorded for the harder bimodal and fully lamellar microstructures. Intermediate force values were observed when cutting the mill annealed sample. On the contrary, no clear relations can be found in the case of feed-per-tooth equal to 0.5 μm/tooth. At this low feed rate, two phenomena can occur affecting the cutting forces: tool run-out and ploughing. When ploughing occurs the material is elastically or plastically deformed, but the chip is not formed by shear as happens in normal cutting conditions [28]. For this reason, the following discussion is mainly focused on the condition of feed rate equal to 1.5 μm/tooth.

It is interesting to observe that the largest scatter of force values was measured in the case of bimodal microstructure, which, as reported in Table 2, shows also the largest difference in hardness values between equiaxed and lamellar grains (Δ_Hardness_ = 130 HV). On the contrary, the scatter of force values was limited when milling the fully lamellar sample, because the microstructure is all made of coarse lamellar grains.

The feed-per-tooth influence on cutting force is obvious: the higher the feed-per-tooth the higher the cutting force due to the increase of the chip section.

Finally, the measuring windows position does not show any influence on the cutting force meaning that the tool has the same behavior from the beginning to the end of the cut.

### 3.3. Tool Condition

**Figure 7:**
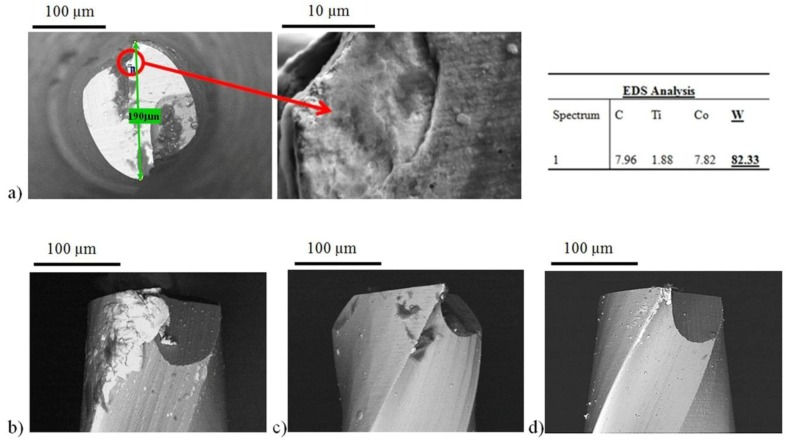
Scanning electron microscope (SEM) images of (**a**) coating delamination (fully equiaxed microstructure) with corresponding energy dispersive spectroscopy (EDS) analysis results; (**b**) built up edge (mill annealed microstructure); (**c**) tool condition after machining fully lamellar microstructure and (**d**) tool condition after machining bimodal microstructure.

The tool condition after machining was evaluated focusing attention on tool damage and built up edge extension. SEM equipped with EDS analysis was used to study the morphology and the chemical composition of the tool surfaces after machining for better understanding of the tool conditions. It was observed that under the considered cutting parameters the main phenomena affecting tool status are abrasion and adhesion. As evident from Figure 7a, material adhesion causes tool coating delamination; in these zones, the EDS analysis highlighted the presence of tungsten carbide and cobalt instead of the coating components (Ti and Al nitrides). As a consequence, the tool substrate comes directly in contact with the workpiece material and the tool damage accelerates.

Another observed phenomenon was the built up edge. Figure 7b shows the picture of the most extended built up edge observed.

In particular, when working fully lamellar material these phenomena are limited (Figure 7c). Once again, this good behavior is mainly related to the presence of coarse lamellae that makes the material more brittle reducing its adhesion and, as a consequence, the built up edge and also to the low hardness of the fully lamellar sample that gives a contribution in reducing the tool damage.

Tool coating delamination and built up edge formation were mainly observed on the tools used to work fully equiaxed and mill annealed microstructures. These microstructures, characterized by higher hardness, show higher tool damage and built up edge (Figure 7a,b) because they are more ductile, having a low volume fraction of lamellar grains with lamellae of small size and, as it is well known, the reduction of lamellae size produces a large increase in ductility [29].

Despite the presence of equiaxed grains with high ductility, the bidmodal microstructure is an exception to this trend, because it shows a limited built up edge (Figure 7d). This can be related to the high hardness difference between equiaxed and lamellar grains measured on this sample, which can have the effect of reducing the material adhesion.

In conclusion, it can be pointed out that the fully equiaxed and mill annealed structures gave the worst results.

**Figure 8:**
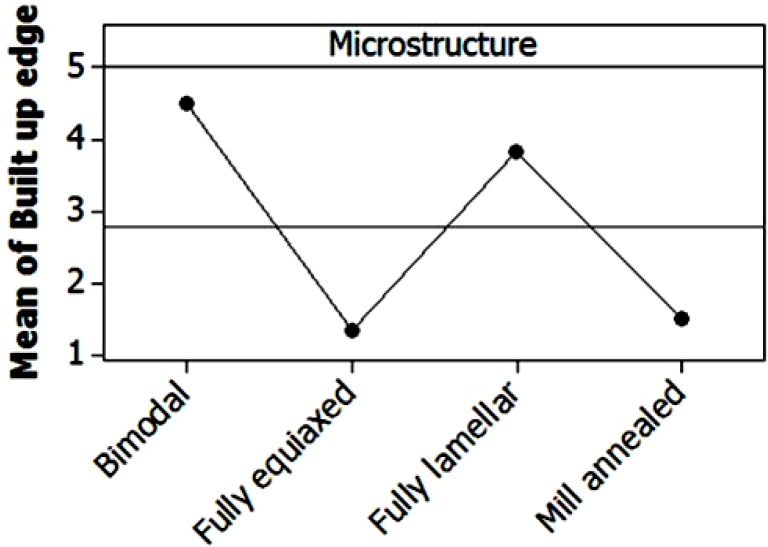
Main effect plot for the tool built up edge index based on SEM images. 1 = poor; 5 = good.

Since it was difficult to correctly quantify the extension of tool damaged area, because of the presence of built up edge, a qualitative tool condition parameter was considered taking into account the built up edge extension. Every tool was thoroughly observed during the SEM analysis in order to provide an evaluation of the qualitative parameter ranging from 1, meaning very extended built up edge, up to 5, meaning absence of built up edge. The ANOVA analysis confirmed the SEM observations. As shown in Figure 8, where the main effects plot for the built up edge is reported, the best results, in terms of built up edge reduction, were obtained when milling bimodal or fully lamellar microstructures.

### 3.4. Channel Geometry and Burr Analysis

To understand the process parameters influence on the quality of the produced parts, the geometry of micro channels and burrs was measured by means of a vision measuring machine (Mitutoyo Quick Scope QS200Z) (Mitutoyo, Kanagawa, Japan).

**Figure 9:**
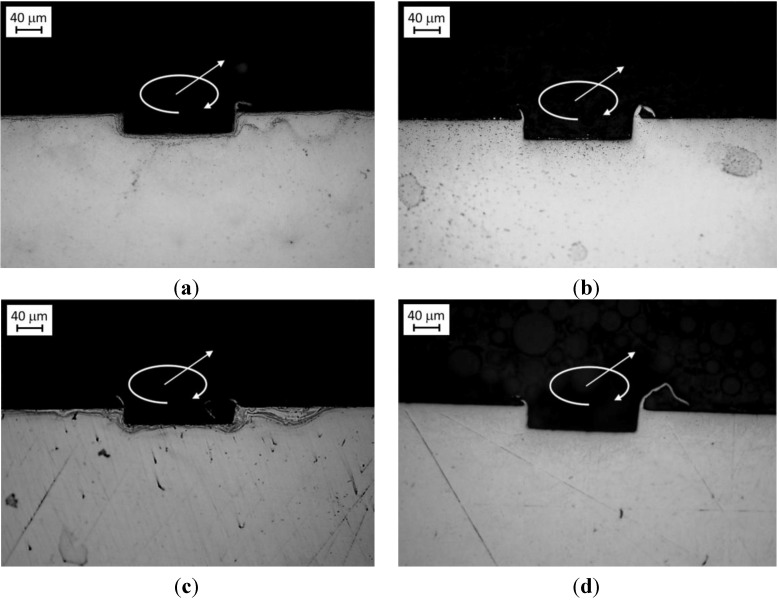
Optical microscope images of micro channel sections (feed-per-tooth 1.5 μm/tooth). (**a**) bimodal; (**b**) fully equiaxed; (**c**) fully lamellar and (**d**) mill annealed. The arrows show the feed and the rotation directions of the mill.

Figure 9 shows the sections of channel realized for a feed-per-tooth equal to 1.5 μm for each microstructure; the same channel shape was observed for all the other cutting conditions. It is evident that no particular differences are noticeable in the channel shape for the different cutting conditions. This is because each channel was realized with a single milling pass using a new mill.

**Figure 10:**
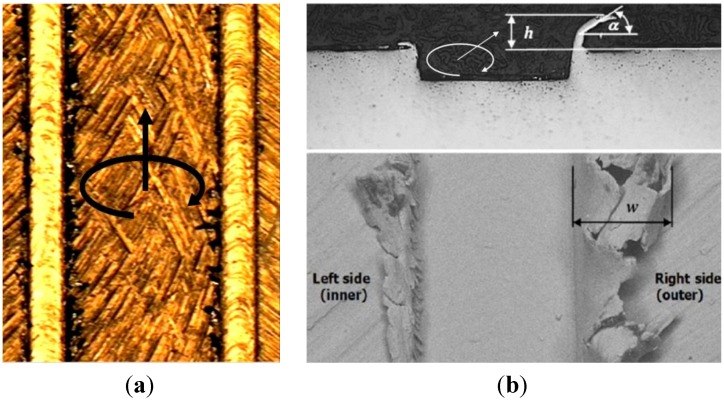
(**a**) Image from vision measuring machine (fully lamellar microstructure, 1.5 μm/tooth); (**b**) Optical microscope image of typical micro channel geometry and SEM image of burr observed from the top. In these pictures the definitions of burr’s width (*w*), height (*h*) and angle (α) are reported. The arrows show the feed and the rotation directions of the mill.

A more evident effect of the microstructure was obtained when considering burrs. As reported in Figure 10, three parameters were considered for defining the burr geometry: the width (*w*), the height (*h*) and the angle (α).

These parameters were measured in two different positions (1 and 2 in Figure 4) equally distributed along the channel length, distinguishing between the right and the left side of the channels. The burr width was measured observing the top surface of the sample (Figure 10a); while for measuring the height and the angle of the burr it was necessary to cut the samples. The sample sections taken in position 1 and 2 were mounted in resin and polished to guarantee a good burr height and angle measurement. Cutting and polishing operations were carefully carried out to maintain the metallographic section perpendicular to the channel. Cold mounting was preferred to hot mounting to avoid the use of the mounting press and subsequent burr deformation.

**Figure 11:**
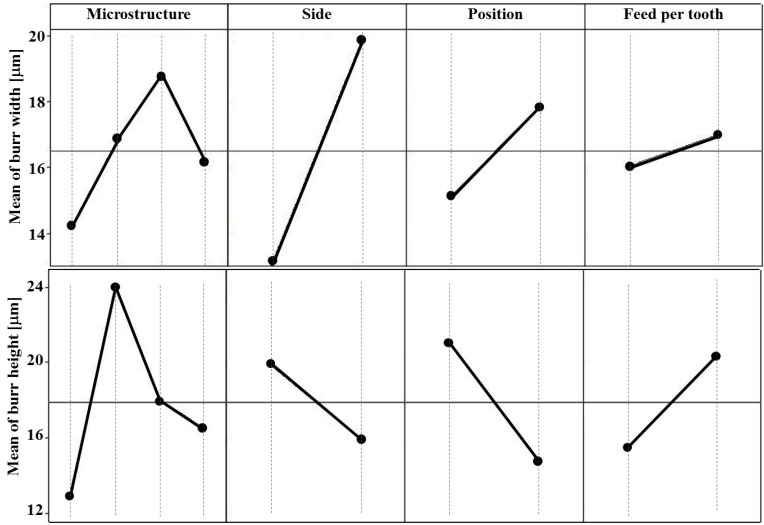

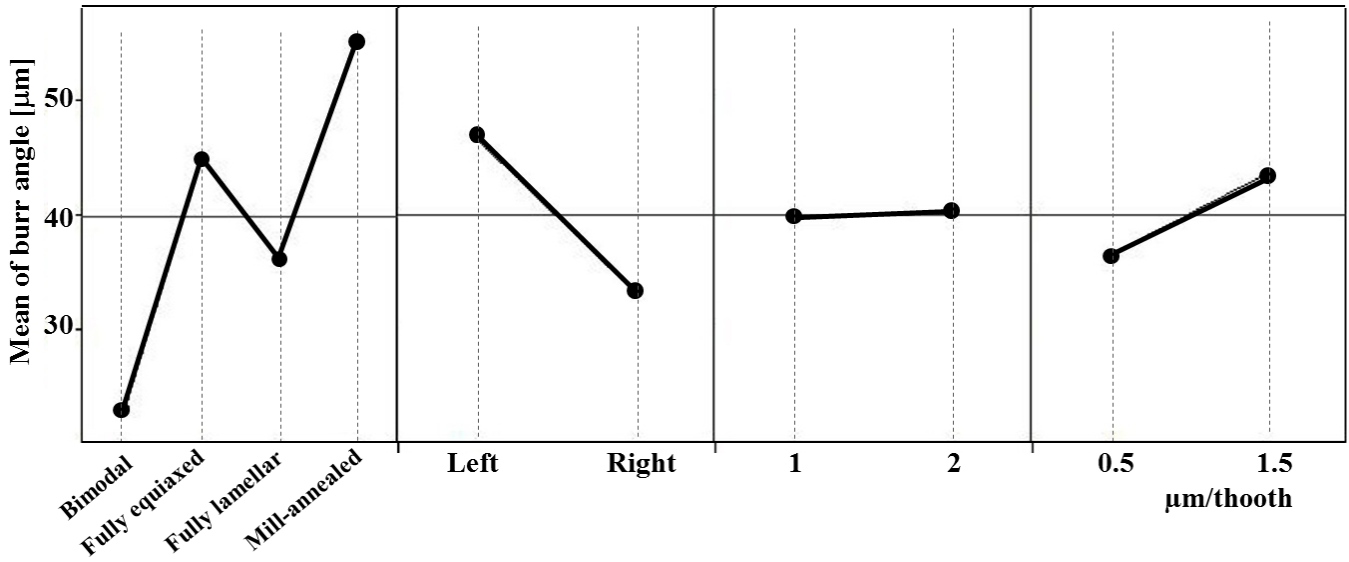
Main effect plots for burr width, height and angle.

Also in this case DOE analyses were carried out in order to understand the process parameters influence (Figure 11) on the burr geometry. The results show that the whole burr geometry is affected by the material microstructure while the burr width depends also on the channel side. The greatest burr width corresponds to the right side of the channel, *i.e.*, the exit side of the cutting edge. The different position (1 and 2 in Figure 4) along the micro channel influences only the burr width and height.

It is evident that the higher the feed-per-tooth, the higher the burr dimensions. When considering the influence of microstructure, the best results were found with the bimodal microstructure, which showed the minimum values for all the considered parameters. No interactions between the considered factors were evidenced.

## 4. Conclusions

In the present paper a study of the influence of the microstructure of Ti6Al4V alloy on cutting force, cutting tool status, and micro channel quality was discussed.

In particular, it has been demonstrated how different microstructures, namely mill annealed, fully equiaxed, fully lamellar and bimodal, affect the flat end micromilling of micro channels when changing the cutting feed. Attention was focused on force detection and analysis, cutting edge status, micro channel aspect and burr formation. It was observed that the material structures affect the process quality. A fully lamellar structure guarantees a better tool status and cutting conditions. In fact, its low hardness reduces cutting forces and tool wear. A coarse lamellae makes the material brittle decreasing its adhesion with the consequent built up edge. Finally, its homogeneous microstructure, without the presence of hard equiaxed α grains, decreases fluctuations in cutting forces.

The micro channel and burr quality are also affected by the material structure. Lower burrs were observed when working a bimodal structure.

The obtained results are very important from a practical point of view since they demonstrate that it is fundamental to correctly select the right material microstructure when micro milling titanium alloy parts. In fact, it is preferable to work material with lamellae instead of equiaxed grains thus obtaining lower and more constant cutting forces and lower tool built up edge together with a higher part quality.
